# Prevalence of sleep disorders in medical students in Uzbekistan

**DOI:** 10.1192/j.eurpsy.2026.11312

**Published:** 2026-07-31

**Authors:** N. D. Kodirov, E. L. Nikolaev, S. A. Yusupov, J. N. Daminov

**Affiliations:** 1 Social and Clinical Psychology Department, I.N. Ulianov Chuvash State University, Cheboksary, Russian Federation; 2 Department of Pediatric Surgery No 1; 3 Department of Pharmacognosy and pharmaceutical technology; 4 Faculty of Pharmacy, Samarkand State Medical University, Samarkand, Uzbekistan

## Abstract

**Introduction:**

Poor sleep quality is a frequent cause of health problems in medical students. The prevalence of sleep disorders in such students, as assessed with the help of the internationally acknowledged PSQI questionnaire, varies approximately from 48.3% in India (Goel et al., 2023) up to 77% in Saudi Arabia (Alotaibi et al., 2020).

**Objectives:**

Our objective was to conduct a PSQI based analysis of sleep quality and prevalence of sleep disorders in medical students of one of Uzbekistan universities.

**Methods:**

Seventy medical students in different years and different branches of learning (60% men and 40% women) aged 18-34 (mean = 22.9, SD =3.2) were asked to complete the self-report Pittsburgh Sleep Quality Index (PSQI) questionnaire. We analyzed the received data using a method of descriptive statistics at the 0.05 significance level.

**Results:**

The majority of the respondents reported sleep disorders (65.71%). Poor quality of sleep is more prevalent in men (63.04%) than in women (36.94%). The mean PSQI global score in the group of medical students in Uzbekistan was 6.53±2.96, which is reliably higher than the standard score (p<0.01). We have also determined the following factors of deterioration of sleep quality according to their intensity as compared with the norm: a reduced duration of sleep (p<0.01), a more frequent use of sleeping pills when required (p<0.01), a more frequent day dysfunction due to sleepiness (p<0.01) and directly disrupted sleep (p<0.01).

**Conclusions:**

The revealed findings prove that the quality of sleep in medical students in Uzbekistan is subnormal, with nearly two out of three students suffering from sleep disorders. Taking the intermediate place among the similar findings received in other countries, these data are not maximal. They also need confirmation after testing a bigger number of respondents in different universities of the country with regard to the students’ gender, year they study in, and academic performance. Nevertheless, these findings might become a basis for planning educational activities and preventive measures in medical universities with a view to improving the students’ sleep quality and preventing any mental and somatic disorders.

**Disclosure of Interest:**

None Declared

